# Evaluation and Validation of Housekeeping Genes as Reference for Gene Expression Studies in Pigeonpea (*Cajanus cajan*) Under Drought Stress Conditions

**DOI:** 10.1371/journal.pone.0122847

**Published:** 2015-04-07

**Authors:** Pallavi Sinha, Vikas K. Singh, V. Suryanarayana, L. Krishnamurthy, Rachit K. Saxena, Rajeev K. Varshney

**Affiliations:** 1 International Crops Research Institute for the Semi-Arid Tropics (ICRISAT), Hyderabad, 502324, India; 2 School of Plant Biology and Institute of Agriculture, The University of Western Australia, 35 Stirling Highway, Crawley, WA, 6009, Australia; University of Delhi South Campus, INDIA

## Abstract

Gene expression analysis using quantitative real-time PCR (qRT-PCR) is a very sensitive technique and its sensitivity depends on the stable performance of reference gene(s) used in the study. A number of housekeeping genes have been used in various expression studies in many crops however, their expression were found to be inconsistent under different stress conditions. As a result, species specific housekeeping genes have been recommended for different expression studies in several crop species. However, such specific housekeeping genes have not been reported in the case of pigeonpea (*Cajanus cajan*) despite the fact that genome sequence has become available for the crop. To identify the stable housekeeping genes in pigeonpea for expression analysis under drought stress conditions, the relative expression variations of 10 commonly used housekeeping genes (*EF1α*, *UBQ10*, *GAPDH*, *18SrRNA*, *25SrRNA*, *TUB6*, *ACT1*, *IF4α*, *UBC* and *HSP90*) were studied on root, stem and leaves tissues of Asha (ICPL 87119). Three statistical algorithms geNorm, NormFinder and BestKeeper were used to define the stability of candidate genes. geNorm analysis identified *IF4α* and *TUB6* as the most stable housekeeping genes however, NormFinder analysis determined *IF4α* and *HSP90* as the most stable housekeeping genes under drought stress conditions. Subsequently validation of the identified candidate genes was undertaken in qRT-PCR based gene expression analysis of *uspA* gene which plays an important role for drought stress conditions in pigeonpea. The relative quantification of the *uspA* gene varied according to the internal controls (stable and least stable genes), thus highlighting the importance of the choice of as well as validation of internal controls in such experiments. The identified stable and validated housekeeping genes will facilitate gene expression studies in pigeonpea especially under drought stress conditions.

## Introduction

Quantitative real-time PCR (qRT-PCR) is one of the most precise, sensible and widely applied techniques to investigate the candidate genes expression [1, 2]. Gene expression based on qRT-PCR profiling depends on the constant performance of housekeeping control genes or simply as reference genes used in a study for normalization of expression of targeted candidate genes [3–6]. These housekeeping genes are essential for normal cell growth and regulation of basic metabolic pathways [7, 8].

A number of housekeeping genes such as ß-actin (*ACT*), glyceral-dehyde-3-phosphate dehydrogenase *(GAPDH)*, 18S ribosomal RNA *(18SrRNA)*, 25S ribosomal RNA *(25SrRNA)*, polyubiquitin *(UBQ)*, ubiquitin conjugating enzyme *(UBC)*, elongation factor 1-A *(EF1A)* and tubulin *(TUB)* etc. have been used as reference genes in different expression profiling studies in many plant species [9, 10]. Nevertheless, there are a number of reports available stating that the expression of housekeeping genes may vary depending on different experimental conditions and crops [11–13]. To select stable reference genes, several studies have been conducted in a number of crop species such as chickpea [10], wheat [14], soybean [15, 16], maize [17], Indian mustard [18], rice [19] and peanut [20]. However, such studies have not taken in case of pigeonpea (*Cajanus cajan* L.).

Pigeonpea is the sixth most important legume food crop which is grown in low-input and risk-prone marginal environments and is often subjected to water stress at different stages of growth and development. Despite having deeper root system, terminal drought is still one of the major factors limiting yield, especially at critical seedling and reproductive stages in pigeonpea [21]. Draft genome sequencing of pigeonpea has provided an excellent platform to study functional expression of any candidate gene(s) which can be utilized for crop improvement [22]. Additionally, a number of transcriptomic resources have been generated in pigeonpea, which could be utilized for the selection of putative candidate genes for gene expression analysis [23–26]. Additionally, through generation of EST libraries and *in-silico* studies few drought responsive genes were identified, which were further validated through qRT-PCR based expression profiling or through transgenic experiments [27, 28]. The gene discovery and marker information gained from pigeonpea genome and transcriptome sequencing have improved pigeonpea genomic resources, which need to be utilized efficiently for crop improvement.

Keeping in view of above, the present study reports comprehensive analysis of 10 commonly used housekeeping genes and identification of the most stable gene(s) for using as internal control for expression studies under drought stress conditions in pigeonpea.

## Results

### Selection of housekeeping genes

A set of ten commonly used housekeeping genes (*EF1α*, *UBQ10*, *GAPDH*, *18SrRNA*, *25SrRNA*, *TUB6*, *ACT1*, *IF4α*, *UBC* and *HSP90*) was selected from different expression studies undertaken in several other crops S1 Table. Homology search of the above mentioned genes has provided their homologs in pigeonpea genome, which were subsequently used for primer designing. As a result, 10 primer pairs were designed for amplifying selected ten genes in pigeonpea for qRT-PCR analysis (Table 1).

**Table 1:** Details of primers used for qRT-PCR analysis.

Gene name	Gene ID	Gene description	Primer sequence 5’ – 3’	Amplicon size (bp)
*EF1α*	B9SPV9	Elongation factor Tu GTP binding domain	F-GAGAGGTCCACCAACCTTGA	103
			R-TTGTAGACGTCCTGCAATGG	
*UBQ10*	Q8H159	Ubiquitin family	F-CCAGACCAGCAGAGGTTGAT	102
			R-GATCTGCATACCTCCCCTCA	
*GAPDH*	Q2I0H4	Glyceraldehyde 3-phosphate dehydrogenase	F-ATGGCATTCCGTGTTCCTAC	95
			R-CCTTCAACTTGCCCTCTGAC	
*18SrRNA*	A5COJ4	18S ribosomal RNA	F-CCACTTATCCTACACCTCTC	102
			R-ACTGTCCCTGTCTACTATCC	
*25SrRNA*	B7FKH8	25S ribosomal RNA	F-ACCCTTTTGTTCCACACGAG	107
			R-GACATTGTCAGGTGGGGAGT	
*TUB6*	B9R897	Tubulin/FtsZ family, GTPase domain	F-GCCCTGACAACTTCGTCTTC	100
			R-GCAGTTTTCAGCCTCTTTGC	
*ACT1*	C6TJ78	Actin 1	F-GGCATACATTGCCCTTGACT	97
			R-GAACCTCGGGACATCTGAAA	
*IF4α*	C6T8X3	Initiation factor 4a	F-GCCGAGATCACACAGTCTCA	95
			R-ACCACGAGCCAAAAGATCAG	
*UBC*	Q2V732	Ubiquitin-conjugating enzyme	F-CGAGAAAAGGCAGTTGATCC	105
			R-CAGAAAAGGCAAGCTGGAAC	
*HSP90*	A5AHA8	Heat shock protein 90	F-TGTCGAGCAAGAAGACGATG	103
			R-GGGCAGTTTCAAAGAGCAAG	

### qRT- PCR amplification efficiencies and expression profiling of housekeeping genes

To find out the stable housekeeping genes, mRNA levels in all the 12 tissues (drought imposed and control tissues) were determined based on their cDNA concentration. The details of 12 tissue samples utilized in the present study are presented in Table 2. In order to compare the expression of all the selected genes across different samples, PCR efficiencies were calculated based on 10-fold serial dilutions of pooled cDNA. Gel electrophoresis of PCR products of cDNA of each individual primers amplified specific size of PCR fragment, with no primer dimer formation indicating the specificity of the primers (S1 Fig). The slopes derived from the measurement of the serial dilutions of cDNA and the PCR efficiency of all the ten genes were found > 90% and its ranged from 90.94 (*IF4α*) to 104.43 (*UBQ10*). The detailed descriptions along with standard and dissociation curve of all the 10 genes used in the present study are presented in S2 Fig and S3 Fig, respectively.

**Table 2:** Description of different tissue samples used for qRT-PCR analysis.

Sample no.	Sample code	Description*
1	EDRC	Early drought root control
2	EDRS	Early drought root stress
3	LDRC	Late drought root control
4	LDRS	Late drought root stress
5	EDSC	Early drought shoot control
6	EDSS	Early drought shoot stress
7	LDSC	Late drought shoot control
8	LDSS	Late drought shoot stress
9	EDLC	Early drought leaf control
10	EDLS	Early drought leaf stress
11	LDLC	Late drought leaf control
12	LDLS	Late drought leaf stress

*Early denotes for vegetative and Late denotes for reproductive stage conditions

The cycle threshold (Ct) values of all the 10 candidate genes for the 12 different samples under study were used to compare the expression rates among themselves and across the different samples. The analysis of datasets revealed a wide range of expression differences between genes. The Ct mean values of selected genes ranged from 8.86 (*25SrRNA* in EDLC: early drought leaf control tissue) to 28.91 (*TUB6* in EDRC: early drought root control tissue).

**Fig 1:**
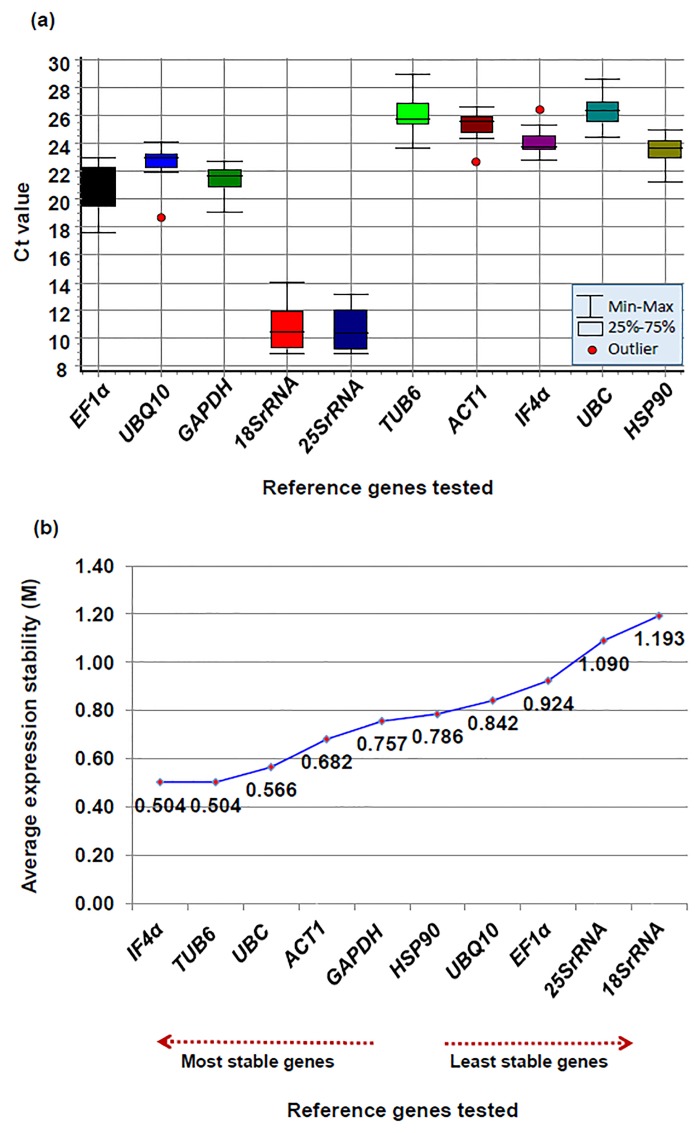
Ct variation and expression stability analysis of each candidate reference gene among different tissue samples using geNorm. (a) Boxplot depicting absolute Ct values, which was calculated using GenEx program. Lower and upper boxes indicate the 25th and 75th percentile, respectively. The median is depicted by the line and all outliers are indicated by dots (b) Gene expression stability graph based on average expression stability values (*M-value*), using stepwise exclusion process. The lower the *M*-*value* indicates, higher the stability of gene. The direction of the arrow indicates the most and least stable housekeeping genes.

**Fig 2:**
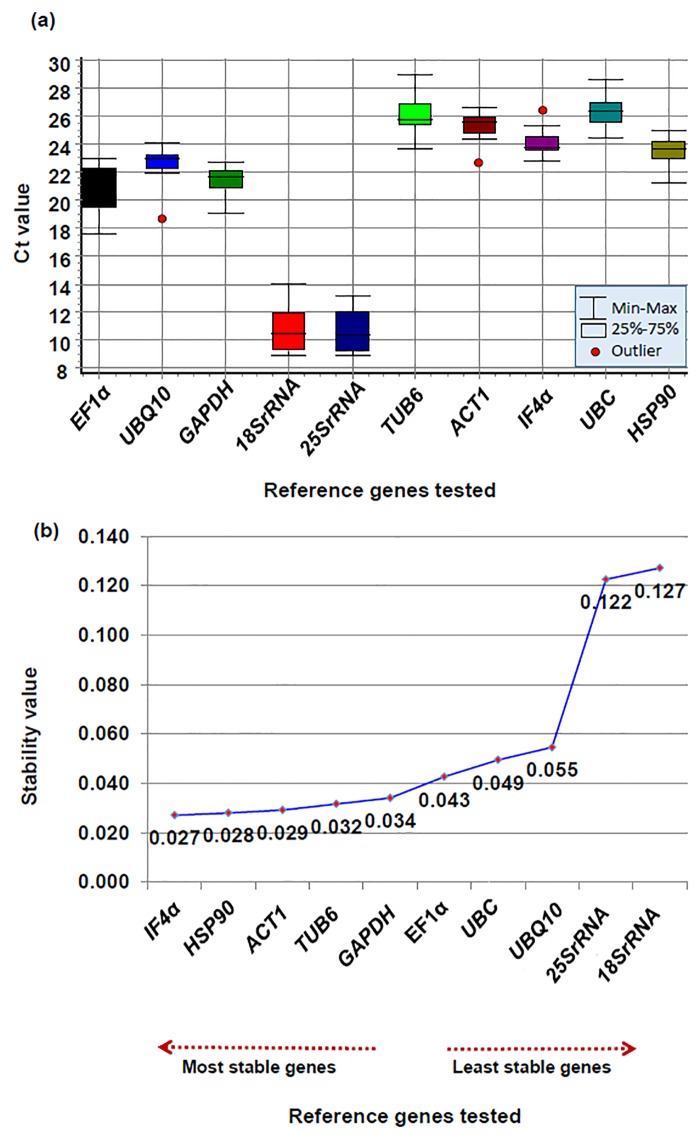
Ct variation and expression stability analysis of each candidate reference gene among different tissue samples using NormFinder. (a) Boxplot depicting absolute Ct values, which was calculated using GenEx program. Lower and upper boxes indicate the 25th and 75th percentile, respectively. The median is depicted by the line and all outliers are indicated by dots. (b) Gene expression stability graph using NormFinder algorithm based on stability values. Lower the stability value indicates higher stability of the housekeeping gene. The direction of the arrow indicates the most and least stable housekeeping genes.

Based on the absolute Ct mean values for each selected gene, *IF4α* and *UBQ10* showed a lower expression variation however, *18srRNA* and *25SrRNA* showed maximum expression variation (Fig 1a and Fig 2a) The Ct mean values of targeted genes were also calculated across the tissues to identify genes with a small level of variations using geNorm (S4 Fig) and NormFinder algorithms (S5 Fig). Even though the variation analysis based on Ct values revealed some of the genes with less variation, however, identification of most stable genes for normalizing gene expression based on different statistical algorithms is necessary.

### Analysis using BestKeeper algorithm

The descriptive statistics of all the ten housekeeping genes used in the study were computed by BestKeeper algorithm [29]. Out of 10 housekeeping genes, 7 (*UBQ10*, *GAPDH*, *ACT1*, *IF4α*, *UBC*, *HSP90* and *TUB6*) showed standard deviation (SD) value ≤1 indicating their consistent and stable performance. The other three genes *25SrRNA* (SD, 1.33), *18SrRNA* (SD, 1.37) and *EF1α* (SD, 1.45) were found inconsistent and showed least stable performance during the analysis. Similarly, the coefficient of variation (CV) of housekeeping genes ranged from 3.15% for *ACT1* to 12.64% for *18SrRNA*, suggesting the presence of different level of variation in target housekeeping genes while analyzing across the tissues (S2 Table).

### Analysis using geNorm algorithm

To determine the ranking of selected housekeeping genes, geNorm algorithm [30] was used to calculate the average expression stability value (*M-value*), using Ct values of each genes across the tissues. Based on the geNorm algorithm, genes with the lowest *M*-*value* were considered as the most stable, whereas genes with the highest *M*-*value* were considered to be the least stable. Nevertheless, it cannot separate between the *M-value* of top two stable genes, so this analysis resulted in the two most stable reference genes in the supplied datasets (Table 3). The average expression stability (*M-value*) of all the tested genes was lower than the 1.5 cutoff established by [30]. The *M-value* of genes ranged from 0.504 for *IF4α* and *TUB6* to 1.193 for *18SrRNA* (Table 3). Thus the *M*-*value* of geNorm analysis identified *IF4α* and *TUB6* as the genes with most consistent expression, whereas *18SrRNA* and *25SrRNA* were found to be the least stable genes (Fig 1b). Based on the Ct mean values of individual genes heat map was developed, which correlates the stability ranking of the identified genes. *IF4α* and *TUB6* showed consistent expression across the tissues while, other genes showed variable levels of expression across the tissues (S6 Fig).

**Table 3:** Ranking of tested housekeeping genes for drought stress conditions using geNorm and NormFinder algorithms.

Factor	geNorm		NormFinder	
	*M-value*	Ranking	Stability value	Ranking
*EF1α*	0.924	7	0.043	6
*UBQ10*	0.842	6	0.055	8
*GAPDH*	0.757	4	0.034	5
*18SrRNA*	1.193	9	0.127	10
*25SrRNA*	1.090	8	0.122	9
*TUB6*	0.504	1	0.032	4
*ACT1*	0.682	3	0.029	3
*IF4α*	0.504	1	0.027	1
*UBC*	0.566	2	0.049	7
*HSP90*	0.786	5	0.028	2

### Analysis using NormFinder algorithm

The stability of selected 10 housekeeping genes was further analyzed using the NormFinder algorithm [31]. The NormFinder analysis of the datasets estimated the stability value of all tested genes based on intra- group and inter- group variation. The genes with less stability values were considered to be the most stable, whereas; with highest stability values were ranked as the least stable genes. Based on the stability value of all genes, *IF4α* (stability value, 0.027) was identified as the most stable gene followed by *HSP90* (stability value, 0.028) (Table 3). The results of the NormFinder were marginally different from that of the geNorm analysis, however, *25SrRNA* (0.122) and *18SrRNA* (stability value, 0.127) were found to be the least stable genes (Fig 2b and Table 3). Heat map analysis of the housekeeping genes revealed that identified stable genes with low stability value, *IF4α* and *HSP90* showed consistent level of expression across the tissues in comparison to the genes with least stability value (*18SrRNA* and *25SrRNA*) (S7 Fig). Therefore, based on geNorm and NormFinder analysis *IF4α* was found to be the most stable housekeeping gene followed by *TUB6* and *HSP90*, which could be used as reference genes for expression analysis of candidate genes under drought stress conditions.

### Validation of identified stable reference genes

**Fig 3:**
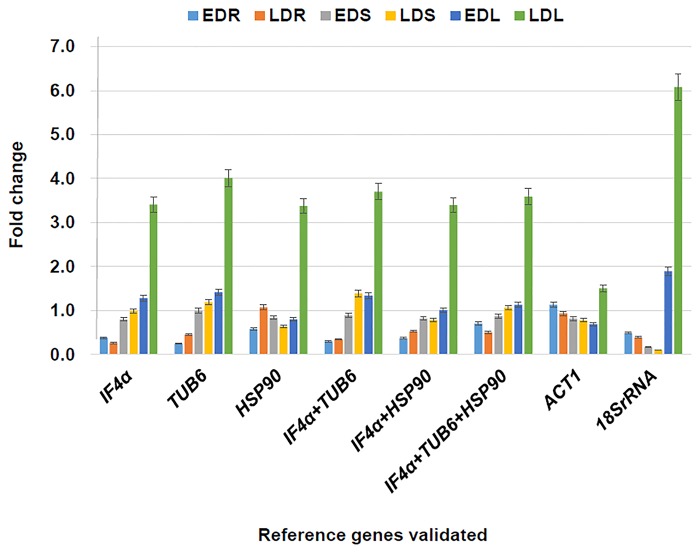
Validation of selected housekeeping genes under drought stress conditions. Expression profiling of candidate gene responsible for universal stress protein A-like (*uspA*) protein in drought imposed tissues (root, stem and leaves). The expression value of candidate gene was normalized with stable, combination of stable and least stable genes namely (i) *IF4α* (ii) *TUB6* (iii) *HSP90* (iv) *IF4α + TUB6* (v) *IF4α + HSP90* (vi) *IF4α + TUB6+HSP90* (vii) *ACT1* and (viii) *18SrRNA*. The relative quantitative values of selected drought responsive candidate gene were obtained after scaling to control samples. EDR: vegetative root stressed; LDR: reproductive root stressed; EDS: vegetative stem stressed; LDS: reproductive stem stressed; EDL: vegetative leaves stressed; LDL: reproductive leaves stressed.

To validate the identified most stable housekeeping genes (*IF4α*, *TUB6* and *HSP90*), combinations of stable housekeeping genes (*IF4α* + *TUB6*, *IF4α* + *HSP90* and *IF4α* + *TUB6* + *HSP90*), least stable housekeeping gene (*18SrRNA*) and the most commonly used internal housekeeping gene (*ACT1*) was used in the normalization of target gene, universal stress protein A-like (*uspA*), to see the expression variability in three different tissues (root, stem and leaves) at two different stress drought conditions (early and late). The relative quantification of the *uspA* gene varied according to the internal controls (stable and least stable genes) used during normalization of the target gene (Fig 3).

Comparative analysis of expression profiling datasets of root tissues at two different stages (EDR: early drought, root stress and LDR: late drought, root stress) revealed a differential level of expression of candidate gene (*uspA*), when normalized with different internal reference genes. Higher levels of candidate gene expression were observed when *ACT1* (1.13 fold for EDR and 0.936 for LDR) is used as an internal control in comparison to the *IF4α* (0.38 fold for EDR and 0.26 fold for LDR), *TUB6* (0.24 fold for EDR and 0.45 fold for LDR) and HSP90 (0.58 fold for EDR and 1.07 fold for LDR). Similar to the expression of the most stable gene individually, expression of stable genes in combination of two such as *IF4α* + *TUB6* (0.30 fold for EDR and 0.35 fold for LDR) and *IF4α* + *HSP90* (0.37 fold for EDR and 0.53 fold for LDR) as well as expression in combination of all three genes i.e. *IF4α* + *TUB6 + HSP90* (0.70 fold for EDR and 0.50 fold for LDR) also showed similar level of expression of the target candidate gene. Not only this, even the least stable gene *18SrRNA* (0.49 fold for EDR and 0.39 fold for LDR) also showed similar level of expression when compared with stable and combination of stable genes (Fig 3).

Analysis of datasets for two different stress conditions of stem tissues (EDS: early drought, stem stress and LDS: late drought, stem stress) revealed expression difference of a targeted candidate gene (*uspA*) when normalized with the most and the least stable genes. Normalization of candidate gene expression data with *ACT1* (0.81 fold for EDS and 0.78 fold for LDS), most stable genes *IF4α* (0.79 fold for EDS and 0.99 fold for LDS), *TUB6* (1.00 fold for EDS and 1.19 fold for LDS) and HSP90 (0.84 fold for EDS and 0.63 fold for LDS). Similarly combinations of stable genes such as *IF4α* + *TUB6* (0.89 fold for EDS and 1.39 fold for LDS), *IF4α* + *HSP90* (0.82 fold for EDS and 0.79 fold for LDS) and *IF4α* + *TUB6* + HSP 90 (0.87 fold for EDS and 1.06 fold for LDS) also showed similarity in expression, while utilizing any of them as internal control. However, comparatively low fold of expression was observed when utilizing least stable gene *18SrRNA* (0.17 fold for EDS and 0.11 fold for LDS) for normalization of targeted candidate gene (*uspA*).

Likewise, analysis of datasets for two different stress conditions of leaves tissues (EDL: early drought leaves stress and LDS: late drought leaves stress) revealed significant differences while utilizing stable and least stable genes in comparison to the most commonly used gene (*ACT1*). Lower level of expression was observed when utilizing *ACT1* (0.69 fold for EDL and 1.50 fold for LDL) as an internal control in comparison to the stable *IF4α* (1.28 fold for EDL and 3.41 fold for LDL), *TUB6* (1.41 fold for EDL and 4.01 fold for LDL) and HSP90 (0.80 fold for EDL and 3.37 fold for LDL). While comparing the fold change in expression of candidate gene, combination of stable genes, *IF4α* + *TUB6* (1.34 fold for EDL and 3.70 fold for LDL), *IF4α* + *HSP90* (1.01 fold for EDL and 3.40 fold for LDL) and *IF4α* + *TUB6* + *HSP90* (1.13 fold for EDL and 3.59 fold for LDL) also showed similarity to the most stable genes. Although, least stable gene *18srRNA* (1.89 fold for EDL and 6.08 fold for LDL) showed a higher level of expression in comparison to the stable, combination of stable genes and most widely used gene *ACT1*.

This finding clearly suggests that the stable genes and combination of stable genes showed similar levels of gene expression in comparison to the commonly used and least stable genes. Therefore, in future combinations of stable genes could be utilized for normalization of candidate genes under drought stress conditions. This study highlights the importance of the choice of internal controls in drought stress conditions for precise identification of candidate gene(s) through expression profiling of candidate genes.

## Discussion

Selection of suitable reference gene is a pre-requisite for expression studies in order to minimize the experimental errors, because an inappropriate reference gene selection can lead to an incorrect interpretation of results [6, 9, 32, 33]. Therefore, to perform the accurate expression analysis of candidate gene(s), number of stable housekeeping genes have been tested and stable genes have been identified in many crops like rice [19, 34], maize [17], wheat [14, 35], potato [36], coffee [37], faba bean [38], soybean [15, 16, 39], peanut [20, 40].

Recent development of genetics and genomics resources [41] and genome sequencing of pigeonpea [22] unveiled the opportunity to precisely identify the candidate genes for various economically important traits, including abiotic stress tolerance in view of abrupt climate change [42]. Till date, very limited studies have been conducted in pigeonpea for identification of drought responsive genes. For instance, through generation of ESTs, transcript profiling and transgenic experiments, three putative candidate genes namely, *C*. *cajan* hybrid-proline-rich protein (*CcHyPRP*), *C*. *cajan* cyclophilin (*CcCYP*) and *C*. *cajan* cold and drought regulatory (*CcCDR*) were identified for abiotic stress tolerance including drought stress [27]. Recently, through *in-silico* comparative analysis between soybean and pigeonpea and validation through qRT-PCR based approaches three drought responsive genes were identified namely, *dehydrin-like protein* (DLP), *acid phosphatase class B family protein* (APB) and *lipid transfer protein 1-like* (LTP1) [28]. However, quantification of identified candidate genes through EST based libraries or through *in-silico* approaches required accurate normalization of the putative candidate genes for further studies. Therefore, to select appropriate reference genes for expression studies of drought stress conditions in pigeonpea, we have analyzed 10 commonly known housekeeping genes on a set of diverse tissues. Three different statistical algorithms were used to analyze the datasets, namely, BestKeeper [29], geNorm [30] and NormFinder [31] for identification of stable reference genes.

BestKeeper algorithm determines the optimal housekeeping gene employing the *pairwise correlation analysis* of all pairs of tested housekeeping genes [30]. Whereas, geNorm algorithm uses step-wise exclusion of the least stable genes, based on the average expression stability (*M*) value which is indirectly proportional to the stability of genes, *i*.*e*. lower the *M*-*value* higher the stability of genes [30]. Therefore, geNorm algorithm provides a pair of ideal housekeeping gene with identical expression ratios regardless of the conditions. NormFinder is an Excel based mathematical modelling algorithm to determine the expression stability value of the gene and to identify the stable reference genes based on intra- and inter-group variation among the tested genes [31]. Ranking of genes obtained by using geNorm and NormFinder may provide different results as they work upon different algorithms [9, 43, 44]. Based on previous studies and algorithms used by these programs, BestKeeper was utilized for analyzing descriptive studies of different housekeeping genes, while geNorm and NormFinder were used to determine the ranking of genes used in the present study [19, 20].

Expression profiling of selected housekeeping genes for drought stress conditions identified *IF4α* as the most stable housekeeping gene followed by *TUB6*, whereas *18SrRNA* and *25SrRNA* were considered as the least stable housekeeping genes. The gene, *IF4α* was also identified to be a stable housekeeping gene across various stress conditions in chickpea [10]. Importantly, this analysis also suggested that housekeeping genes like *18SrRNA* and *25SrRNA* [45] should be avoided as an internal control in expression profiling studies for drought stress conditions in pigeonpea. The housekeeping gene *IF4α* (Initiation factor 4a) identified in the present study is known to be a RNA helicase and the prototype of the DEAD-box family of proteins, which are involved in a variety of cellular processes including splicing, ribosome biogenesis and RNA degradation [46, 47].

Two most stable (*IF4α* and *TUB6*), combination of stable genes (*IF4α* + *TUB6*), widely used (*ACT1*) and least stable gene (*18SrRNA*) were used as an internal control to evaluate the comparative expression variation of the candidate gene in different tissues and developmental stages. The expression analysis of the datasets revealed that the expression of stable genes (*IF4α* and *TUB6)* alone and in combination (*IF4α* + *TUB6*), showed a relatively similar level of expression, while utilize to normalize the expression level of the target candidate gene (Fig 3). However, during the analysis under drought stress conditions higher level of gene expression was observed in leaves tissue (LDL; reproductive leaves stressed) as compared to stem and root. To get insight into this, a detailed study needs to be done in future. This analysis also revealed that most widely used internal gene (*ACT1*) and least stable gene (*18SrRNA*) showed significant levels of expression differences between stable genes for one or two tissue specific stages. Validation of identified stable reference genes using targeted candidate gene, increases the reliability of the results for expression analysis of candidate genes. Therefore, it can be concluded that stable genes identified through ranking of the housekeeping genes, utilizing different algorithms can be used as an internal control in expression profiling studies under drought stress conditions.

## Conclusions

To the best of our knowledge this is the first study on identification of stable housekeeping genes in pigeonpea, which could be used as an internal control in gene expression studies for drought stress conditions. A total of 10 candidate housekeeping genes was selected and evaluated in 12 different samples for drought stress conditions (early and late) using the three different algorithms namely, BestKeeper, geNorm and NormFinder. Analysis of datasets revealed set of stable housekeeping genes, which were further validated using previously identified candidate gene for drought stress conditions. These identified and validated stable housekeeping gene(s) could be used as internal control for wider applications of gene expression studies in pigeonpea.

## Materials and Methods

### Plant material and growth conditions

In the present study, Asha (ICPL 87119) a leading pigeonpea variety was selected for gene expression studies. The genetically pure seeds of Asha were obtained from Pigeonpea Breeding division, ICRISAT, Patancheru which was developed by crossing C11 × ICP1-6-W3/W. Seeds were thoroughly washed with DEPC treated water and pre-soaked overnight. Germinated seedlings were sown in the center of 3 inch plastic pots (one per pot) filled with autoclaved black soil, sand and vermi-compost (10:10:1 v/v) mixture. All the plants were grown under controlled glass-house conditions in three biological replicates [48]. Fresh tissues (root, stem and leaves) were harvested from two different growth stages (vegetative and reproductive) and stored immediately in liquid nitrogen till RNA isolation. A total of 12 pigeonpea tissues were collected from three plant parts, *i*.*e*., root, stem and leaves, covering two developmental stages, vegetative and reproductive stage under drought stress conditions (Table 2).

### Drought stress treatments

Slow drought (dry down) stress was imposed for 45 days-old (vegetative stage) and 75 days-old-plants (reproductive stage). Calculated amount of water was added to each of the pots and was weighed regularly. Control plants were maintained at 80% of relative water content (RWC) throughout, whereas stressed plants were dried down up to 20% RWC.

### RNA isolation and quality controls

Total RNA was isolated using TRIzol reagent (Invitrogen, USA) following manufacturer’s instructions. RNA samples were purified using DNase (Qiagen, GmbH, Germany) through an RNeasy Plant Mini kit according to the manufacturer’s instruction (Qiagen, GmbH, Germany). The integrity of RNA samples was assessed on 0.8% agarose/formaldehyde gel electrophoresis. The concentration of each sample was checked on the Qubit fluorometer (Invitrogen) and three micrograms of RNA was used for first-strand cDNA synthesis using the SuperScript III RT enzyme (Invitrogen, USA) following the manufacturer’s guidelines.

### qRT-PCR primer designing and test of amplification efficiency

Ten commonly known housekeeping genes (Table 1) were selected and their pigeonpea orthologous sequences were used for primer designing. For functional integrity, BLASTN search against GenBank EST database was performed (pigeonpea database reference). Primer pairs were designed from exonic regions utilizing Primer3 software (http://probes.pw.usda.gov/cgi-bin/batchprimer3/batchprimer3.cgi). Quantitative real-time PCR (qRT-PCR) was performed using ABI SYBR GREEN PCR reaction on an ABI Fast7500 System (Applied Biosystems [ABI], Foster City, CA) according to the manufacturer’s instructions. The amplification efficiency of primers was estimated by SYBR Green chemistry RT-qPCR assay using 1, 10^-1^, 10^-2^, 10^-3^ and 10^-4^ fold dilutions of pooled cDNAs of three technical replicates for each gene. PCR conditions used for all qRT-PCR were: 2 min at 50°C, 10 min at 95°C, and 40 cycles of 15 s at 95°C and 1 min at 60°C. Each reaction was performed in three biological and two technical replicates along with no template control. Amplicon specificity was verified by melting curve analysis and agarose gel electrophoresis.

### Gene expression stability analysis

Expression stability of 10 selected housekeeping genes (*EF1α*, *UBQ10*, *GAPDH*, *18SrRNA*, *25SrRNA*, *TUB6*, *ACT1*, *IF4α*, *UBC* and *HSP90*) over three tissues (root, shoot and leaves) under drought stress conditions comprising of total 12 samples were analyzed using BestKeeper descriptive statistical method [29]. This is Microsoft Excel based software freely downloadable from http://download.gene-quantification.info/. It identifies most suitable genes using repeated pairwise correlation and regression analysis of a given gene with all other candidate housekeeping genes.

The ranking and identification of most suitable genes for given conditions, statistical algorithms geNorm and NormFinder were used. The geNorm (http://medgen.ugent.be/~jvdesomp/genorm/) algorithm is based on the principle of average expression stability value or *M-value* of housekeeping genes and it eliminates the gene with high *M-value* and repeats the process until there are only two genes left, which are identified as the two most stable genes. The remaining pair of housekeeping genes is recommended as the optimum number of housekeeping genes required for normalization of qRT-PCR datasets [30]. Another algorithm, NormFinder uses linear mixed-effects modelling to calculate stability values to find out the optimum number of reference genes to be used for normalization of qRT-PCR datasets [31]. This is a Microsoft Excel based free program (http://moma.dk/normfinder-software), for the identification of stable housekeeping genes.

### Validation of identified reference genes

For validation of identified reference genes root, shoot, and leaves tissues were collected from drought stressed conditions. A drought responsive universal stress protein A-like (*uspA*) coding gene, selected from our earlier studies (data, unpublished) was used to validate the expression level of most stable, combination of most stable, least stable, and commonly used reference genes. RNA isolation and qRT-PCR were performed as mentioned previously and relative expression level was measured using a Relative Expression Software Tool (REST©) [49]. Expression levels of drought stressed samples were compared to their respective unstressed controls and checked for their differential expression using different reference genes.

## Supporting Information

S1 Fig Amplification of a specific PCR product with cDNA. Agarose gel (2%) showing amplification of specific PCR products of expected size for each 10 housekeeping genes tested in the present study.
(TIF)

S2 Fig Standard curve for the real-time PCR. The X-axis represents the log10 cDNA dilution series, and the Y-axis represents the cycle threshold (Ct). The reaction efficiency (E) is given by [10^(1/-S)^-1] × 100%, where S represents the slope of the linear regression line.
(TIF)

S3 Fig Specificity of real-time PCR amplifications. Dissociation curves for ten housekeeping genes with single peak obtained from two technical replicates of 12 different cDNA pools. X-axis represents temperature (°C) and Y axis represents Derivative reporter (-Rn).
(TIF)

S4 Fig Gene expression analysis of candidate housekeeping genes across the tissues based on geNorm algorithm. This figure shows Ct distribution of each candidate reference gene among the 12 samples calculated through geNorm algorithm.
(TIF)

S5 Fig Gene expression analysis of candidate housekeeping genes across the tissues based on NormFinder algorithm. This figure shows Ct distribution of each candidate reference gene among the 12 samples calculated through NormFinder algorithm.
(TIF)

S6 Fig Heat map of candidate genes based on geNorm algorithm. This figure shows a heat map of candidate genes plotted based on Ct mean values. Clustering of genes was based upon the Ct mean values of individual candidate genes across tissues. The detailed description of samples is provided in Table 2.
(TIF)

S7 Fig Heat map of candidate genes based on NormFinder algorithm. This figure shows a heat map of candidate genes plotted based on Ct mean values. Clustering of genes was based upon the Ct mean values of individual candidate genes across tissues. The detailed description of samples is provided in Table 2.
(TIF)

S1 Table List of stable housekeeping genes identified for selected studies. This table shows list of stable housekeeping genes identified under different biotic and abiotic stress conditions in different crops.
(DOC)

S2 Table Descriptive statistics of candidate housekeeping genes. This table shows descriptive statistics of all 10 candidate housekeeping genes used in the study for drought stress conditions using BestKeeper algorithm.
(DOCX)
