# Supplementary figures and images for: Evaluation and Validation of Housekeeping Genes as Reference for Gene Expression Studies in Pigeonpea (Cajanus cajan) Under Drought Stress Conditions
Source: PLoS One. 2015 Apr 7;10(4):e0122847. doi: 10.1371/journal.pone.0122847 (PMC4388706; doi:10.1371/journal.pone.0122847)

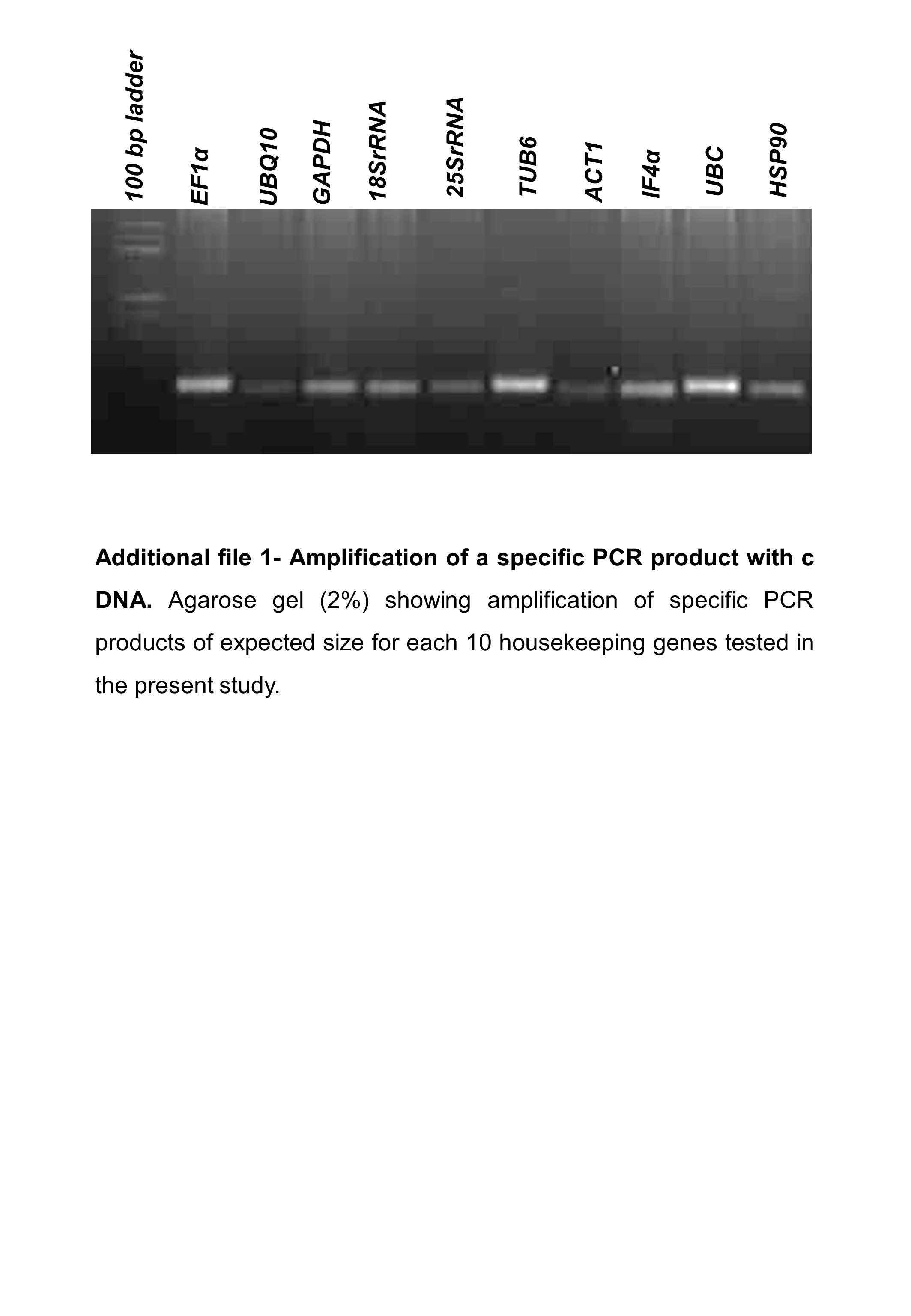

Supplement: S1 Fig — Agarose gel (2%) showing amplification of specific PCR products of expected size for each 10 housekeeping genes tested in the present study. (TIF) [file pone.0122847.s001.tif]

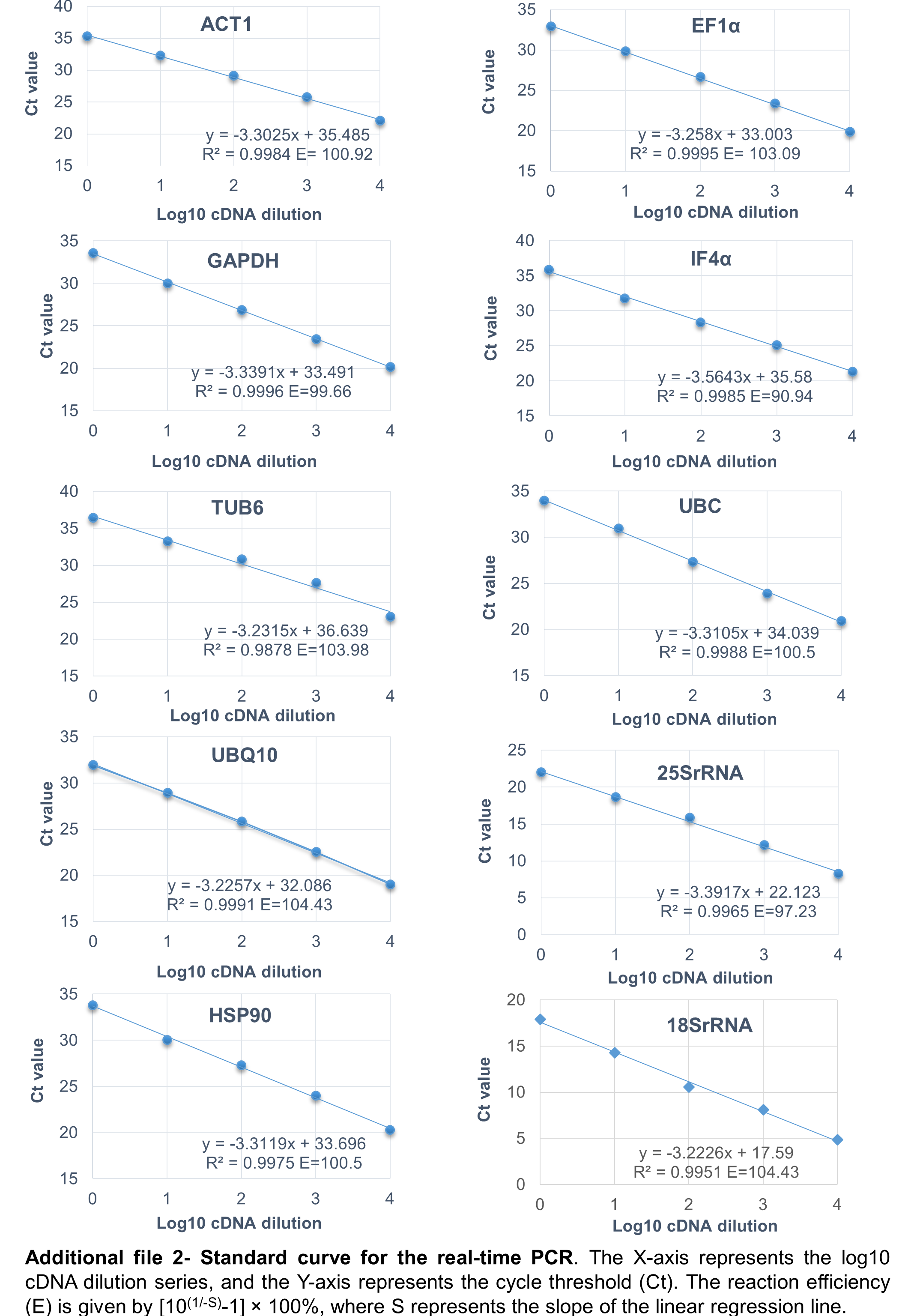

Supplement: S2 Fig — The X-axis represents the log10 cDNA dilution series, and the Y-axis represents the cycle threshold (Ct). The reaction efficiency (E) is given by [10(1/-S)-1] × 100%, where S represents the slope of the linear regression line. (TIF) [file pone.0122847.s002.tif]

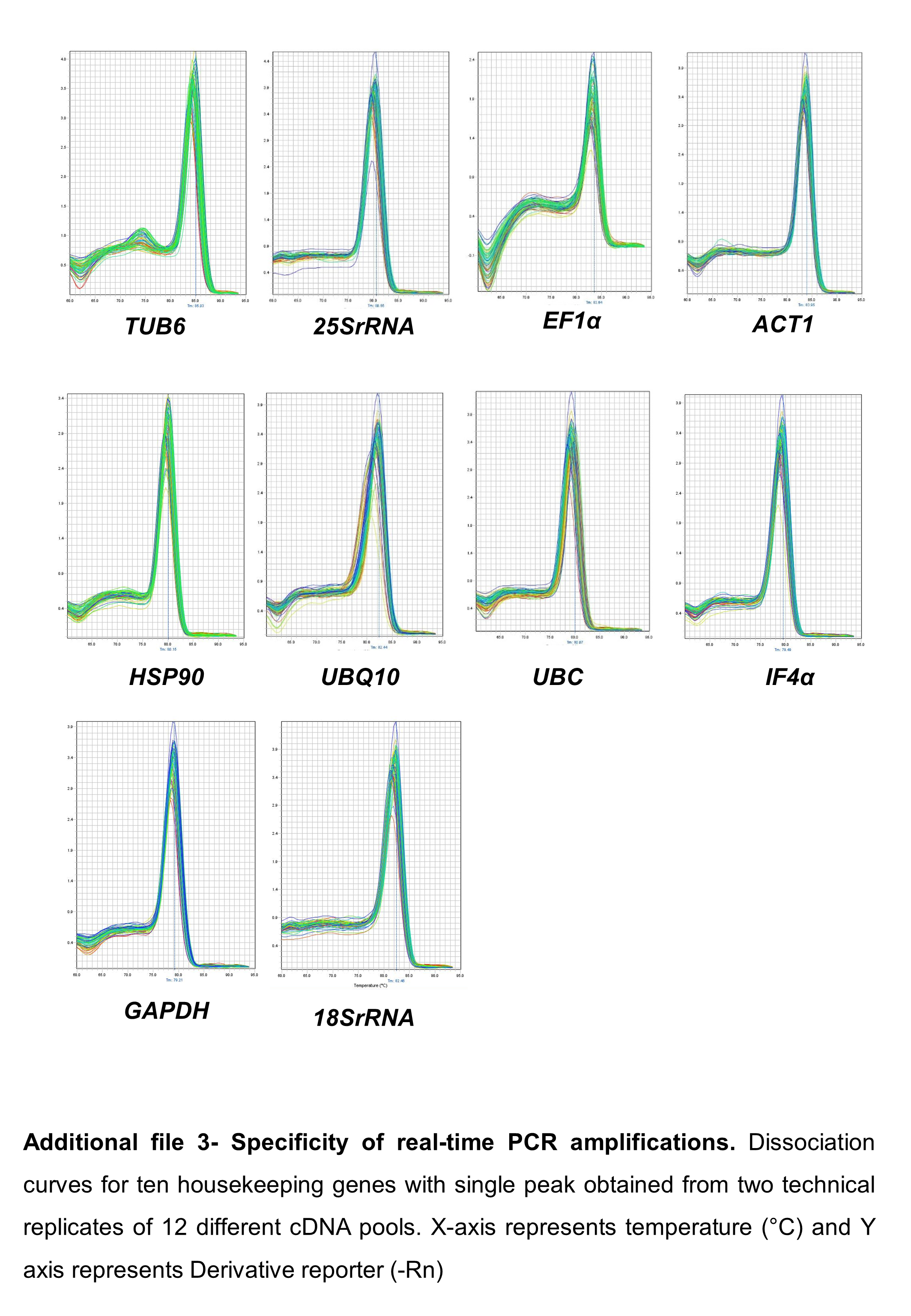

Supplement: S3 Fig — Dissociation curves for ten housekeeping genes with single peak obtained from two technical replicates of 12 different cDNA pools. X-axis represents temperature (°C) and Y axis represents Derivative reporter (-Rn). (TIF) [file pone.0122847.s003.tif]

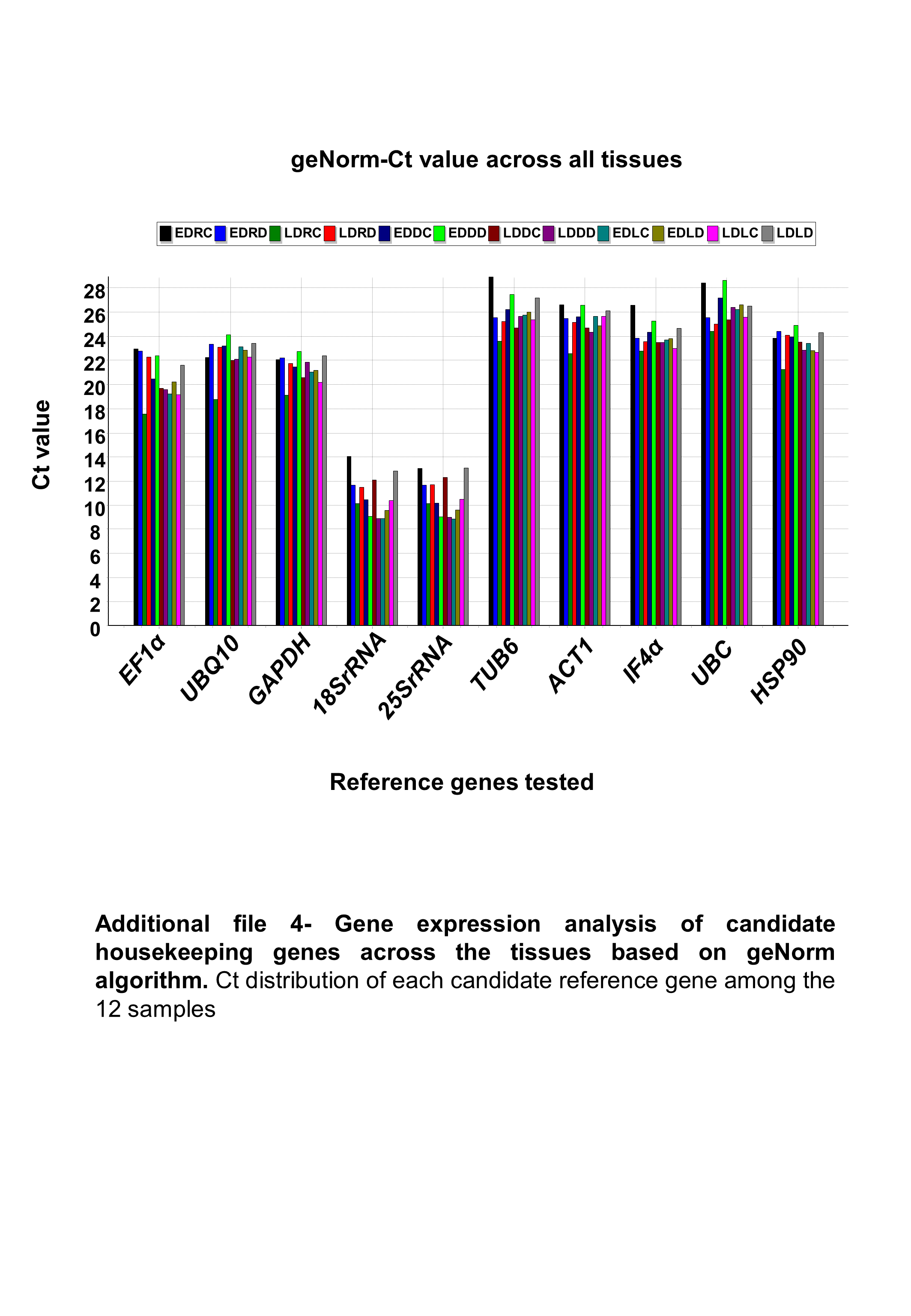

Supplement: S4 Fig — This figure shows Ct distribution of each candidate reference gene among the 12 samples calculated through geNorm algorithm. (TIF) [file pone.0122847.s004.tif]

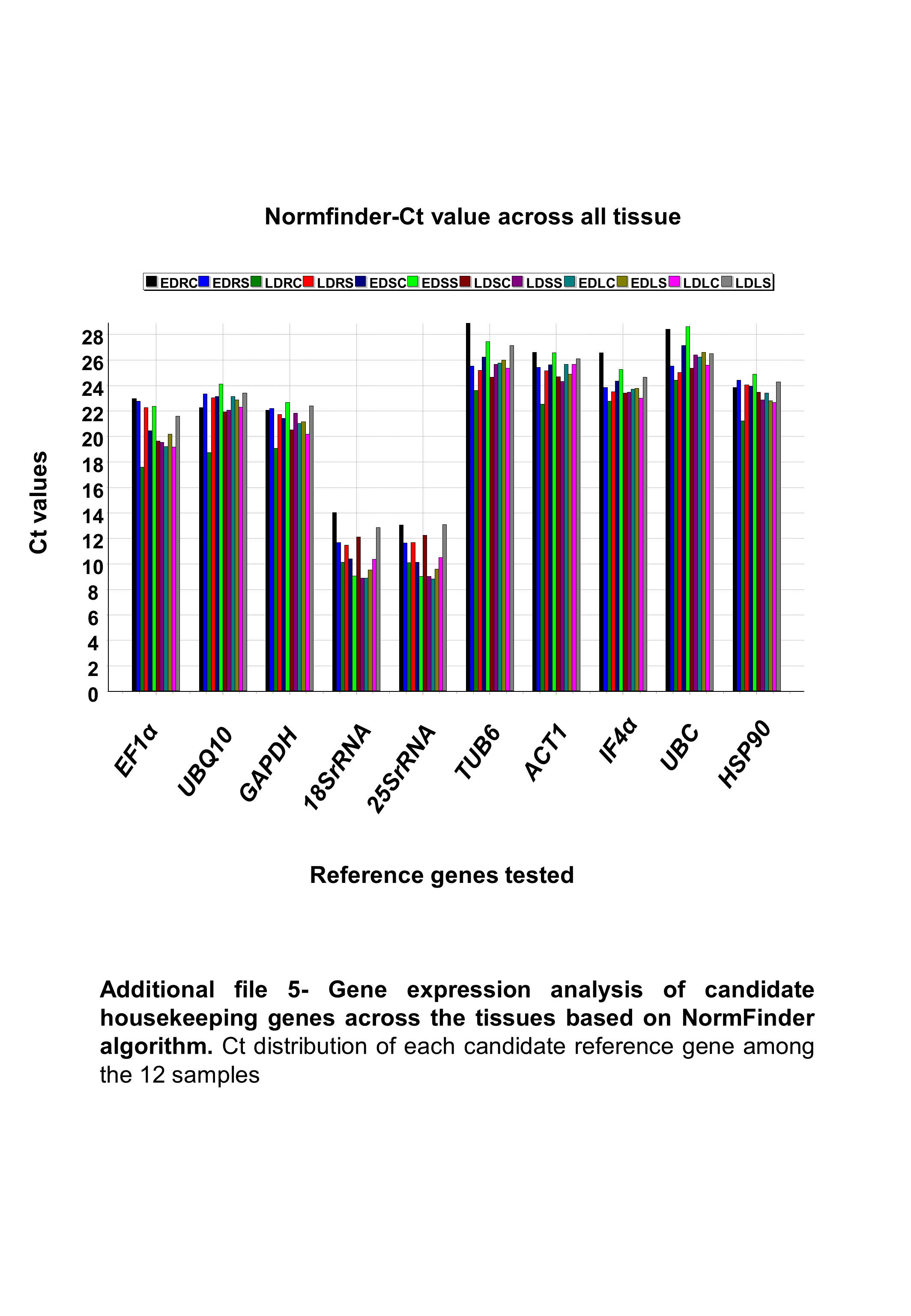

Supplement: S5 Fig — This figure shows Ct distribution of each candidate reference gene among the 12 samples calculated through NormFinder algorithm. (TIF) [file pone.0122847.s005.tif]

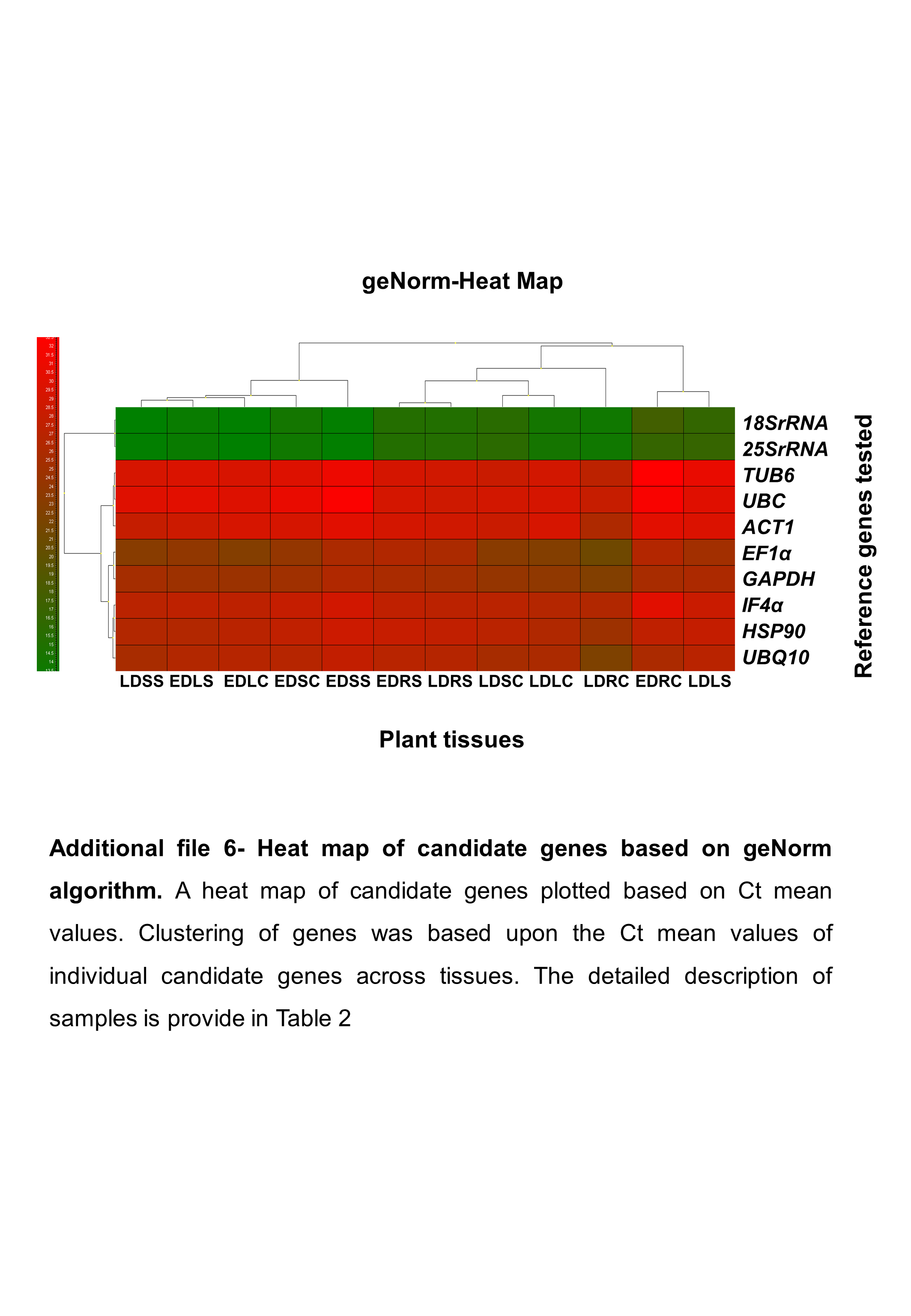

Supplement: S6 Fig — This figure shows a heat map of candidate genes plotted based on Ct mean values. Clustering of genes was based upon the Ct mean values of individual candidate genes across tissues. The detailed description of samples is provided in Table 2. (TIF) [file pone.0122847.s006.tif]

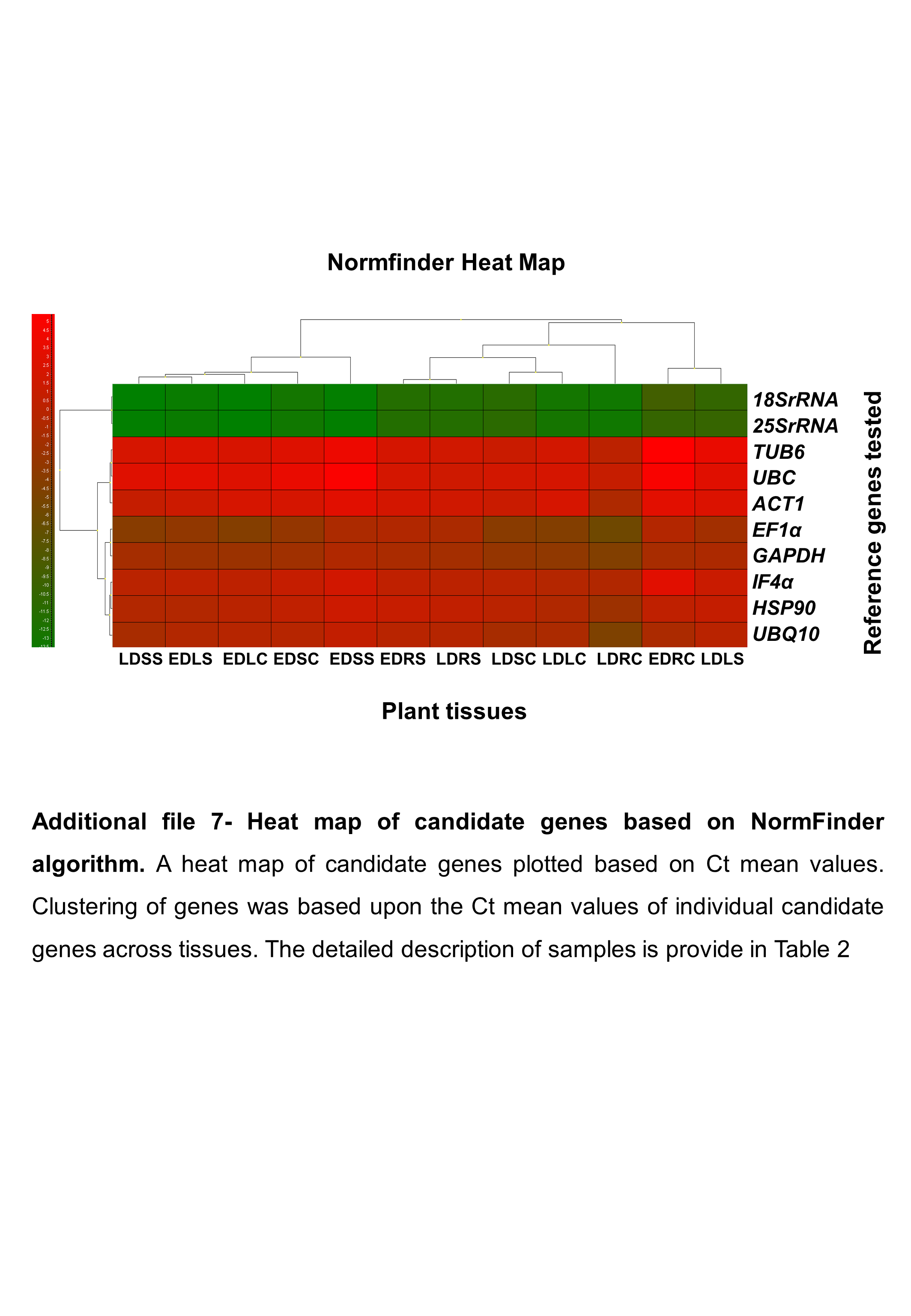

Supplement: S7 Fig — This figure shows a heat map of candidate genes plotted based on Ct mean values. Clustering of genes was based upon the Ct mean values of individual candidate genes across tissues. The detailed description of samples is provided in Table 2. (TIF) [file pone.0122847.s007.tif]
