# Supplementary material for: Evaluation and Validation of Housekeeping Genes as Reference for Gene Expression Studies in Pigeonpea (Cajanus cajan) Under Drought Stress Conditions
Source: PLoS One. 2015 Apr 7;10(4):e0122847. doi: 10.1371/journal.pone.0122847 (PMC4388706; doi:10.1371/journal.pone.0122847)
Supplement: S1 Table — This table shows list of stable housekeeping genes identified under different biotic and abiotic stress conditions in different crops. (DOC) [file pone.0122847.s008.doc]

**Table S1**. List of stable housekeeping genes identified for selected studies

| **Crop** | **Stress** | **Genes used** | **Genes selected** | **Softwares used** | **Reference** |
| --- | --- | --- | --- | --- | --- |
| Chickpea | Drought, salt and cold | 12 genes (*ACT1, EF1α, GAPDH, IF4a, TUB6, UBC, UBQ5, UBQ10, 18SrRNA, 25SrRNA, GRX, HSP90*) | Across developmental stages: *EF1a, HSP90*  Across various stress conditions: *IF4a, GAPDH* | geNorm | Garg et al. (2010) |
| Wheat | Temperature stress (cold and heat) | 12 well-known HKGs  and 20 genes novel with reference to the normalization issue | Gene encoding for *PDI-*like protein | geNorm, NormFinder | Paolacci et al. (2009) |
| Wheat | NaCl, ABA, PEG, low temperature and yellow rust | Genome-wide transcriptome data analysis | *Ta.10105.1.S1_at, Ta.14126.1.S1_at, Ta.1532.1.S1_a_at, Ta.24713.1.A1_at, Ta.27922.1.S1_at, Ta.28753.2.S1_a_at, Ta.3006.1.S1_s_at, Ta.3064.1.S1_x_at, Ta.7156.1.S1_at, Ta.7800.1.A1_at, Ta.7894.3.A1_at,Ta.10763.1.S1_at, Ta.23834.1.S1_at, Ta.28553.3.S1_at* and *TaAffx.97910.1.S1_at* | geNorm, NormFinder | Long et al. (2010) |
| Soybean | 116 biological samples, representing tissues at various developmental stages | Seven commonly used (ACT2, ACT11, TUB4, TUA5, CYP, UBQ10, EF1b) and seven new candidates (SKIP16, MTP, PEPKR1, HDC, TIP41, UKN1, UKN2) | Overall: ACT11, UKN1 and UKN2  Developmental stages: SKIP16, UKN1 and MTP  Photoperiod: TIP41, UKN1 and UKN2 | geNorm | Hu et al. (2009) |
| Soybean | Dehydration, high salinity, cold and ABA | 13 genes (*60s, ABC, Act11, Act27, CDPK, CYP2, ELF1a, ELF1b, Fbox, IDE, SUBI2, TUBa, TUBb*) | Dehydrated root and shoot tissues: *Fbox and 60s*  Salt tress: 60s/Fbox  Cold stress: IDE/60s and Fbox/Act27  ABA: 60s/ELF1b, ELF1b/Fbox and 60s/ELF1b | Delta CT and geNorm | Le et al. (2012) |
| Soybean | Darking, salinity, drought and SMV infection | 8 genes (*ACT11, TUA5, CYP, EF1B, TUA4, TUB4, EF1A* and *ACT2/7*) | Salinity stress: *t EF1A, ACT11,* Drought stress: *TUB4, TUA5, EF1A* Dark treatment: *ACT11, UKN2,* Virus infection: *EF1B,* *UKN2* | geNorm, NormFinder | Ma et al. (2013) |
| Maize | High-temperature and dark stress | 12 genes (*UBCE, GRP2, UBCP, FPGS, LUG, UCH, CUL, PGM, MEP, ACT, TUB*) | *CUL, FPGS, LUG, MEP*and*UBCP* | geNorm  NormFinder, BestKeeper | Manoli et al. (2012) |
| Mustard | NaCl, ABA, MeJa, SA, glucose, IAA, ACC and drought | 12 genes (*ACP, ACT2, CAC, ELFA, GAPDH, SNF, TIPS-41, TMD, TSB, TUA, UBQ9, ZNF*) | developmental stages: Combination of *GAPDH, TUA, TIPS-41, CAC*  Various stress and hormone: *UBQ9 and TIPS-41* | geNorm, NormFinder | Chandna et al. (2012) |
| Rice | IAA, Ebr, BAP, ABA, GA3, ACC, SA, JA, CHX, **s**alt and drought | 10 genes (*18S rRNA, 25S rRNA, UBC, UBQ5, UBQ10, ACT11, GAPDH, eEF-1a, eIF-4a, b-TUB*) | Across tissue: *UBQ5, eEF-1a*  Under various environmental conditions: *18S, 25SrRNA* | geNorm | Jain et al. (2006) |
| Rice | Drought, salt, and cold stress | *20* genes (*ABP, APR, EP, EP2, EP3, GAP, GRP, HNR, NBP, VPC, SKR, TRP, TBC, ZCF61, ZF, EF1α, GAPDH, GBP, TPI, UBQ5*) | *EP, HNR* and *TBC* | geNorm, NormFinder | Maksup et al. (2013) |
| Potato | Biotic (late  blight) and abiotic stresses (cold and salt stress) | 7 genes (b-tubulin,  cyclophilin, actin, elongation factor 1-a (*Ef1a*), 18S  rRNA, adenine phosphoribosyl transferase (aprt), and  cytoplasmic ribosomal protein L2) | *Ef1a* | geNorm | Nicot et al. 2009 |
| Peanut | Tobacco streak virus (TSV), Peanut bud necrosis virus (PBNV), Late Leaf Spot (LLS), rust, salinity and drought | 8 genes (*ADH3, ACT11, ATPsyn, CYP2, ELF1B, G6PD, LEC and UBC1*) | *ADH3, G6PD, ELF1B* | geNorm, NormFinder | Reddy et al. (2013) |
| Faba bean | A wide set of samples, including different tissues,  genotypes and several inoculations for the most  important pathogens | 11 genes (*ACT1,*  *ACT11, CYP2,*  *ELF1A,*  *ELF1B, G6PD, TUA, TUB,*  *UBC2, UBQ10* and  *GAPDH*) | *ACT1, CYP2 or ELF1A* | geNorm and NormFinder | Gutierrez et al. (2011) |
