# Supplementary material for: Evaluation and Validation of Housekeeping Genes as Reference for Gene Expression Studies in Pigeonpea (Cajanus cajan) Under Drought Stress Conditions
Source: PLoS One. 2015 Apr 7;10(4):e0122847. doi: 10.1371/journal.pone.0122847 (PMC4388706; doi:10.1371/journal.pone.0122847)
Supplement: S2 Table — This table shows descriptive statistics of all 10 candidate housekeeping genes used in the study for drought stress conditions using BestKeeper algorithm. (DOCX) [file pone.0122847.s009.docx]

**Table S2**. Descriptive statistics of candidate genes using BestKeeper software for drought stress conditions

| Factor |  | Candidate gene | | | | | | | | | |
| --- | --- | --- | --- | --- | --- | --- | --- | --- | --- | --- | --- |
|  |  | *EF1α* | *UBQ10* | *GAPDH* | *18srRNA* | *25srRNA* | *TUB6* | *ACT1* | *IF4α* | *UBC* | *HSP90* |
| GM |  | 20.60 | 22.51 | 21.35 | 10.69 | 10.65 | 25.94 | 25.26 | 24.02 | 26.30 | 23.49 |
| AM |  | 20.66 | 22.55 | 21.38 | 10.80 | 10.75 | 25.97 | 25.28 | 24.04 | 26.33 | 23.51 |
| Min |  | 17.58 | 18.78 | 19.10 | 8.89 | 8.86 | 23.62 | 22.54 | 22.78 | 24.40 | 21.23 |
| Max |  | 22.97 | 24.14 | 22.71 | 14.04 | 13.08 | 28.91 | 26.61 | 26.55 | 28.63 | 24.91 |
| SD |  | 1.45 | 0.89 | 0.80 | 1.37 | 1.33 | 1.00 | 0.80 | 0.78 | 0.97 | 0.74 |
| CV |  | 7.01 | 3.95 | 3.74 | 12.64 | 12.38 | 3.83 | 3.15 | 3.24 | 3.70 | 3.16 |
| Min [x-fold] |  | -8.09 | -13.29 | -4.77 | -3.46 | -3.46 | -4.99 | -6.57 | -2.36 | -3.73 | -4.77 |
| Max [x-fold] |  | 5.17 | 3.10 | 2.56 | 10.20 | 5.41 | 7.85 | 2.56 | 5.79 | 5.04 | 2.68 |
| SD [± x-fold] |  | 2.73 | 1.85 | 1.74 | 2.58 | 2.52 | 1.99 | 1.74 | 1.72 | 1.96 | 1.67 |

Abbreviations: GM: geometric mean; AM: athematic mean; Min and Max: threshold values; SD: standard deviation; CV: coefficient of variation; Max [x-fold] and Min [x-fold]: threshold expression levels expressed as absolute x-fold over or under regulated coefficient; SD [± x-fold] standard deviation of absolute regulation coefficient
